# An Overview of the Importance of Conformational Flexibility in Gene Regulation by the Transcription Factors

**DOI:** 10.1155/2009/210485

**Published:** 2010-02-04

**Authors:** Shagufta H. Khan, Raj Kumar

**Affiliations:** Department of Basic Sciences, The Commonwealth Medical College, Scranton, PA 18510, USA

## Abstract

A number of proteins with intrinsically disordered (ID) regions/domains are reported to be found disproportionately higher in transcription factors. Available evidences suggest that presence of ID region/domain within a transcription factor plays an important role in its biological functions. These ID sequences provide large flexible surfaces that can allow them to make more efficient physical and functional interactions with their target partners. Since transcription factors regulate expression of target genes by interacting with specific coregulatory proteins, these ID regions/domains can be used as a platform for such large macromolecular interactions, and may represent a mechanism for regulation of cellular processes. The precise structural basis for the function of these ID regions/domains of the transcription factors remains to be determined. In the recent years there has been growing evidence suggesting that an induced fit-like process leads to imposition of folded functional structure in these ID domains on which large multiprotein complexes are built. These multiprotein complexes may eventually dictate the final outcome of the gene regulation by the transcription factors.

## 1. Introduction

Biologically functional proteins and/or protein domains/regions that appear to exist as an ensemble of reversible conformers with only little or no well defined secondary/tertiary structures, and are being recognized to contain amino acid sequences that fail to automatically fold into their fully compact functional conformations under physiological conditions, have grown exponentially in last decade or so [1–10]. These are often known as intrinsically disordered (ID) proteins, which possess protein surfaces with largely unstructured and dynamic conformations [1–10]. One common characteristic of many ID protein regions is high number of charged amino acids and low hydrophobicity, which acts to destabilize an ordered conformation [11]. Importance of ID regions/domains in cell signaling and regulation can be easily judged by the fact that these ID containing regions/domains are reported to be much higher in eukaryotic genomes when compared with prokaryotes [12–15]. The abundance of such ID protein regions/domains in eukaryotes could be due to the fact that their flexible and dynamic conformation promotes recognition of target molecules or functional binding partners, by creating large interaction surfaces suitable for macromolecular interactions [9–11]. There are reports showing that transcription factors with modular structures commonly possess one or more of ID regions/domains, and it is believed that nature has created such flexibility for specific functions that may require large structural flexibility under physiological conditions [12, 16].

In spite of having common characteristics of ID nature, these regions/domains often do not share sequence homology with other members and are quite variable in size compared to other similar domains within the transcription factors [17–20]. For example, steroid receptors, which possess an ID activation domain located in their N-terminal domain, are quite variable in size and sequence homology [17]. Due to unstructured nature of these ID domains it has been quite difficult to study their three-dimensional structures, and only in the recent years, we have begun to understand their structural basis [21–23]. However, compared to proteins with globular structures, still not much is known about their three-dimensional structures. As we have begun to understand their physical and functional characteristics, it is now well accepted that in order to function optimally, these ID regions need to acquire well defined conformations under physiological conditions [24–26]. To fully understand how precisely a transcription factor transmits the signal to regulate the expression of its specific target gene(s), it is pivotal to gain structural and functional information about ID regions, particularly those within the activation domain [27]. It is likely that conformational flexibility of ID region allows it to adopt protein surfaces such that an efficient interaction can be established with other target binding partners that can result in ID sequences to achieve ordered conformation(s) to carry out their functions [1–7, 28–32]. The obvious questions then become, what causes these conformations? Is there any unifying mechanism that dictates these conformational alterations? Do all such ID regions adopt a unique conformation under physiological conditions? Are the conformational transitions taking place during folding/unfolding of these ID regions, highly dynamic process? How do internal and external factors influence their structural dynamics in a particular cellular environment? These and several other fundamental questions warrant an answer to understand this complex yet extremely important phenomenon with far reaching biological consequences. Therefore it is important to address the underlying structural and functional correlations that govern this critical, yet not fully understood process.

Some studies have shown that transcription factors remodel chromatin structure in an extremely dynamic situation such that they have the capacity to rapidly form and reform multiprotein complexes involving critical coregulatory proteins including those from the fundamental initiation complex machinery [17]. Thus, the role of their ID region/domain(s) with flexible conformations becomes much more important, and in fact this could provide a mechanism for inclusion or exclusion of specific protein complexes that may ultimately influence the final outcome responsible for regulation of target gene either through activation or repression [33–36]. It is now well accepted fact that transcriptional regulation is a highly complex and dynamic process that allows relatively small number of transcription factors to generate a huge variety of gene expression through various permutations and combinations of their interactions with target binding partners [37–45]. Thus, the notion that ID domains/regions of transcription factors must have significantly ordered conformation in their normal cellular milieu under physiological conditions pose a paradox that must be solved before we can fully understand their role in gene regulation [17]. Hypothetically, there could be several possibilities to resolve this phenomenon [17]. Several research groups including ours are actively involved to answer these questions. It is important to note that under physiological conditions, there are several cellular events including molecular crowding, both due to the presence of small molecules and/or macromolecules that could influence the structure formation in such ID proteins [25]. Since transcription factors function through interactions with a network of gene assembly at the level of protein expression, these kinds of conditional folding become much more relevant for them [5]. In the recent years, data from both experimental and computational approaches are supporting this theory, and are helping us in gaining knowledge about the functional structures of ID proteins/peptides [5, 6]. In this paper, we have discussed various ways by which these ID regions/domains of the transcription factors could acquire functionally ordered conformation(s), essential for their optimal functions under physiological conditions.

## 2. Factors Responsible for Bringing Ordered Conformation(s) in the ID Region/Domain of the Transcription Factors

For years, we have been relying on the theory that according to thermodynamic hypothesis of Anfinsen, the amino acid sequences can provide all information needed to determine the fold of a protein, and only one collapsed folded state is possible for a specific sequence [46]. Until recently, no data challenged this concept; however, more and more we learn about characteristics of protein folding process, it becomes clearer that it is not possible to predict with certainty the folded form of a protein from its primary sequence, and recent progresses made with the class of proteins that are ID or contain ID regions seem to challenge this hypothesis [1–7]. In our opinion, the biggest drawback with such a theory is that it does not consider the highly dynamic and mobile nature of protein conformation that gives such a profound flexibility to protein to adopt a number of conformations in a given cellular environment in a rapid manner. ID proteins can be divided into two classes (at least); those that can adopt a unique native conformation when subjected to stabilizing conditions (i.e., the addition of natural ligands such as proteins and DNA or stabilizing osmolytes), and those that apparently have no stable native state [7, 17]. The ID regions of many transcription factors fall into the category of those ID proteins that can fold into unique structures, suggesting that if functional properties of the transcription factors are coupled to folding/unfolding in the ID region, thermodynamic analysis of the equilibrium should provide a quantitative characterization of their function [7, 12, 34]. In this situation, the change in free energy should be reflected to favor folding process due to the factors responsible for folding [47]. In fact, we and others have discovered several ways to make the ID portion of several transcription factors to fold into a functionally active form that can facilitate transcriptional activity of related protein [47–53]. Thus, the knowledge we have gained so far supports the idea that the conditional binding/folding of the ID regions of the transcription factors may be an important requirement for its role in gene regulation [17].

Since transcription factors work in a very selective manner to regulate specific sets of genes, conformational flexibility of ID region/domain may set up appropriate assembly of coregulatory proteins in an efficient and selective manner to regulate the target gene [17]. It is no secret that ID stretches are quite common in proteins with essential basic cellular functions (including many transcription factors), and thus may be recognized as a separate functional and structural entity based upon the basis of structure and function within the protein classes [1–3]. However, it may be premature to do so, unless more structural and functional characterization of such proteins becomes available. Due to their differential structural and functional characteristics from those of ordered proteins, ID proteins require special experimental and computational tools for their characterization [1, 3]. In a number of signaling proteins, sites of posttranslational modifications (such as site-specific phosphorylation) are located within ID region/domain [6, 54]. One of the main reasons for such propensity is to facilitate extensive formation of hydrogen binding between the backbones and/or side chains that can occur through disorder-order transition within the ID region [54]. The structural flexibility of ID proteins helps them to more easily and specifically adapt to protein:protein and protein:DNA interaction cascades and possibly in gene regulation including alternative splicing [55]. Knowledge of these factors and the kind of conformations adopted by the ID regions/domains within the transcription factors will lead to an understanding of the role of order/disorder transition in the transcription process. Details of some of the possible events that might lead to functionally folded conformations in the ID regions/domains under physiological conditions are discussed in the sections below in detail.

## 3. Osmolyte-Induced Folding of Intrinsically Disordered Region/Domain of the Transcription Factors

Organic osmolytes are found widely in nature to protect cellular proteins against harsh conditions such as the effects of dehydrating conditions, other hypertonic states, or the build-up of potentially denaturing metabolites, and are known to interact with the peptide backbone of proteins [56–69]. Osmolytes are synthesized by microorganisms, plants, and animals in response to environmental stress to protect proteins against denaturation [56–69]. The free energy of these interactions corresponds to the propensity of protein to either fold or unfold due to presence of osmolytes [64–66]. In spite of small energy magnitude of such interactions, peptide bonds are by far the most numerous structural component of a protein [62–64]. Consequently, the sum of such interactions can be quite large [64]. It is the balance between osmolyte-backbone interactions and amino acid side chain-solvent interactions that determines the outcome on protein folding [64]. In most cases, ID regions do not contain sufficient hydrophobic residues to fold spontaneously, thus addition of an osmolyte shifts the balance to a favorable negative free energy for folding [64]. The unfavorable interaction of the osmolyte with the peptide backbone causes the preferential exclusion of the osmolyte from the protein-water interface, and it dominates over any favorable interaction of the osmolyte with the side chains of amino acids of the protein [61]. It is the balance between osmolyte-backbone interactions and amino acid side chain-solvent interactions that determines the outcome on folding [59]. The quantity of osmolyte required depends on both its inherent solvophobic interaction with peptide backbone and the free energy balance provided by the sum of all backbone-osmolyte interactions and the sum of all amino acid side chain-solvent interactions [68, 69].

It has recently been shown that the effects of differing osmolytes are additive, so that under physiological conditions, cellular profolding molecules (such as osmolytes) may reach even higher summative concentrations [64–66]. Certain plants, animals, and microorganisms have adapted to environmental stresses that change the intracellular water activity by producing small organic osmolyte molecules [61, 62]. Indeed, in some organisms and in mammalian cells, certain class of osmolytes arise to counteract the effects of high intracellular concentrations of urea or other denaturing conditions on the biological activity of relevant proteins [61, 62]. Osmolytes are known to perform vital functions in many different tissues in the human body, particularly kidney and brain. Without presence of relatively large quantities of osmolytes, the kidneys may not be able to function [56, 61]. For example, urea tends to decrease the *k*_cat_ and increase the *K*_*m*_ of enzymatic reactions, while the counteracting osmolyte TMAO tends to have the opposite effects, that is, increasing *k*_cat_ and decreasing *K*_*m*_ [70]. Furthermore, it has been shown that urea and osmolyte, trimethylamine-N-oxide have opposite effects alone or in combination [64]. It is therefore logical to believe that many other osmolytes could have similar effects depending upon the environmental conditions and cellular effects. Studies undertaken in last several years from various laboratories on osmolytes suggest that osmolyte-induced structures are in fact native-like with functional activities under physiological conditions [61–64]. Osmolytes are natural substances, used by many organisms to enhance proper protein folding [61–64]. Human kidney, for example, contains several osmolytes, and it has been calculated that osmolyte concentrations in whole tissues often reaches quite high relative to cell water content, suggesting that in certain cells/tissues, their concentrations are almost surely much higher [61, 62].

It is well accepted fact that when a protein folds into a cooperative manner, it should result in a native-like functional species, and the consensus is that when cooperative folding in the presence of an osmolyte occurs, it is to the native folded structure [61, 62]. Osmolytes can force ID protein to fold into native-like functional species with significant secondary and tertiary structural contents in it [17]. Our published data on the osmolyte-induced folding of ID activation domain of steroid receptors strongly supports this idea [17]. We have used several osmolytes to cooperatively fold an ID activation region (AF1) located in the N-terminal domain of the glucocorticoid receptor [71]. We have shown that when AF1 is incubated in increasing concentrations of natural organic osmolytes representative of three classes: certain amino acids (proline), methylamines (sarcosine), and polyols (sorbitol), the ID AF1 peptide folds into functionally active conformation(s) that selectively binds several critical coregulatory proteins, and subsequent transcriptional transactivation activity [71]. A study has shown that oral administration of an osmolyte, trehalose can inhibit polyglutamine-mediated protein aggregation in cerebrum of transgenic mouse model of Huntington disease and increased life span [72]. It has been suggested that these beneficial effects of trehalose are due to stabilizing the partially unfolded polyglutamine-containing Huntingtin protein [72]. This protein aggregation/misfolding process constitutes a hallmark of neurodegenerative pathologies, including Alzheimer's, Huntington's, and Parkinson's diseases, and if osmolytes can provide a unifying mechanism of action, this may have far reaching consequences in developing better therapeutic tools for the management of such diseases. Such effects of osmolytes on protein folding pathways have become important to study. Under physiological conditions, the cellular compositions of osmolytes may vary significantly; therefore, different protein folding pathways utilized in the cell may depend upon the cellular environment within it [61]. Understanding the role of osmolytes in cell regulation will not only allow to predict the action of osmolytes on macromolecular interactions in stressed and crowded environments typical of cellular conditions, but will also provide insights on how osmolytes may be involved in pathologies or in their prevention.

## 4. Role of Site-Specific DNA Binding in the Induced Folding of Intrinsically Disordered Region/Domain of the Transcription Factors

It is well established that to regulate transcription, transcription factors act on specific genes by binding to regulatory element sites in the DNA, generally located upstream from the relevant transcription start site, and termed as response element [27]. Once bound to its specific response element through high affinity and specificity for the relatively short DNA sequences contained therein, the DNA bound transcription factor collects a variety of other coregulators that modify chromatin structure and/or interact with the proteins from the primary transcription initiation complex to regulate transcription from the relevant promoter [73, 74]. Thus, both protein-DNA and protein:protein recognition are central processes in transcription factors function, and several reports indicate that these interactions are often accompanied by conformational changes leading to folding of the ID region(s) in a protein molecule [21, 73, 74]. There are reports that DNA binding stabilizes the overall global fold of protein in a manner that is consistent with folding-coupled target recognition as a mechanism to control site-specific recombination, and protein flexibility is involved in such induced-fit recognition particularly in ID DNA binding proteins [75]. It is an established fact that transient interactions between transcription factors and site-specific DNA sequences are common and fundamental to many cellular processes, and protein flexibility is found to play a major role in protein:DNA binding where conformational flexibility of protein acts to maximize efficiency of protein:DNA binding [75]. For transcription factors, protein first binds DNA nonspecifically (with low affinity) in a partially or fully unfolded state and undergoes folding of ID sequences when it finds specific DNA site to which it binds tightly with high affinity [75].

An important biological implication of this binding/folding phenomenon is that in early events protein backbone mobility may play an important role in a specific binding with target molecule; whereas later events may lead to specific signals being passed to the target gene(s) from the complex of proteins, which emerges only after appropriate conformational changes take place [12]. Based on these observations, it is logical to hypothesize that site-specific nucleotide sequence of the regulatory element sites affects not only the overall affinity of the transcription factor for its regulatory element site, but also influences its overall conformation such that the ID region(s) of these proteins can acquire much needed ordered conformation(s) [17]. As a result of such events ID surfaces on the protein molecule can be modified to accommodate various critical ancillary factors [73, 74]. Since transcriptional regulation for a specific gene depends upon the interactions of these coregulatory proteins, the exact DNA sequences of the available sites in the regulatory region of the DNA of the gene could help determine gene regulation [73, 74]. Biophysical studies (using Circular Dichroism and Fluorescence Emissions) carried out by us have shown that stoichiometric binding to a consensus response element of the glucocorticoid receptor (an intracellular transcription factor, belonging to the nuclear hormone receptors superfamily) results in a considerable amount of binding energy being devoted to intramolecular rearrangement in its N-terminal domain where a powerful ID transactivation domain is located [49]. Similar studies from other groups using the progesterone receptor have also been reported that its site-specific DNA binding results in additional structure in its ID N-terminal domain [52]. Together, these results suggest that one of the reasons why sequence specific DNA binding has such a profound effect on function of the transcription factors in general and the steroid receptors in particular may be so that their ID sequences may acquire an ordered conformation(s) [17].

Since many transcription factors possess ID activation domain that is responsible for their transcriptional transactivation activity, and this activation domain provides a platform for interaction with other coregulatory proteins, DNA binding induced conformational alterations in transcription factors is of immense importance in regulating the expression of target genes [5, 12, 17]. Of course, conformational changes in other parts of the molecule cannot be ruled out. For example, in case of the steroid receptors, DNA-binding induced structural changes in the N-terminal ID domain may be influenced by other intramolecular cross communications such as interactions between N- and C-terminal domains, and/or due to binding of specific ligands [17]. Though these studies certainly provide a reasonable explanation of why such a specific protein:DNA interaction takes place in a promoter region involving transcription factor, resolution of these models will require further future experiments. Of course, availability of three-dimensional structure of such ID containing region bound to DNA through their DNA binding domain will provide much needed information. Since gene regulation is an essential function in all organisms and provides the ability to respond to signals that reflect intra- and extra-cellular environmental conditions, understanding the role of protein:DNA interactions involved in the regulation of gene expression has been a major challenge. In the recent years, a broad range of techniques have been used to explore the molecular and energetic basis of DNA recognition, assembly, and allosteric changes within regulatory proteins that involves transcription factors.

## 5. Role of Protein:Protein Interactions in Giving ID Region a Functionally Folded Conformation

**Figure 1:**
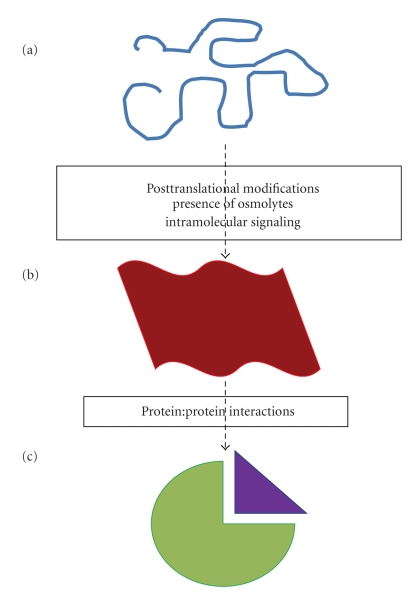
A model showing possible events/factors that may alter conformation of an ID domain/region of transcription factors to fold into a functionally active form. (a) An ID domain/region without any defined structure ((a) blue) may adopt a set of partially folded conformation ((b) red) due to specific events/factors shown here. This conformation may suit well for specific interactions with binding partners (purple) and result in functionally active conformation ((c) green) under physiological conditions.

It is a well established fact that there are a number of proteins often known as coregulatory proteins that make physical and functional interactions with DNA-bound transcription factors and participate in their transcriptional activation function [17]. These coregulators act as coactivators or corepressors depending upon the up- or down-regulation of the target gene by specific transcription factor [17]. Of course, addition of several additional cofactors cannot be ruled out that may be involved either directly or indirectly; some of them are ubiquitous, while others cell-specific [17]. In fact, for many transcription factors, it has been reported that their effects on transcriptional activity may be cell- and promoter-specific and potential explanation for these effects can be attributed to the formation of the assembly of transcription factor with other coregulatory proteins in a particular cellular setup [17]. Thus, specific combination of transcription factor and coactivators/corepressors results in the specific control of particular genes [17]. But the obvious questions then come to mind: how is the choice of coregulator interaction with specific transcription factor made? Some of the explanation for this can be provided from the fact that differing surfaces of the transcription factor are important for regulation of various genes [17]. There are several reports showing that protein:protein interactions may result in induced-fit alterations in the structure formation in ID region of the transcription factors [5]. In Figure 1, we have illustrated a model of binding/folding for ID domains/regions under physiological conditions.

Many ID regions are known to undergo to more ordered conformational transition after interacting with their protein binding targets [5]. For example, ID kinase-inducible transcriptional-activation domain (KID) of CREB folds into a more ordered conformation on binding to its target peptide in CBP [5]. ID activation domain of c-Myc (another transcription factor known to regulate the transcription of genes involved in normal cell growth, differentiation, and apoptosis) selectively binds to proteins from the basal transcription factors, and undergoes induction of protein conformation in the ID domain of c-myc during this interaction with the target factor [29]. Similar studies have been shown involving the activation domain and its target protein for other transcription factors [30–32]. We and others have shown that when an ID regulatory region of steroid receptor binds to its coregulatory protein from basal transcription machinery, structure is formed in the ID activation domain of the steroid receptor [27, 29]. Dyson and Wright have suggested that the binding of ID regions to their targets is often regulated by covalent modifications, which leads to simple biological switches [5]. Applied to the ID region of transcription factors, this induced-fit model of folding hypothesizes that these ID regions do not adopt fully ordered conformation(s) until they bind to one or more of their key target partner proteins [17]. It appears that many transcription factors need to be more flexible in order to be efficient in carrying out their functions [12]. Because specific region(s) of these transcription factors act by interacting with specific binding partner proteins, it is likely that their flexible structure helps them create a favorable surface for these interactions [5, 12].

In the recent years, much attention has been focused on the role of protein:protein interactions in gene regulation by transcription factors, although a systematic analysis of all possible interactions and underlying mechanisms is still lacking. Due to varied expression patterns, many cell types contain an assortment of different factors that can interact with a single transcription factor [17]. It seems likely that the precise protein assembly, based on relative affinities combined with the allosteric effects, largely define the final transcriptional potential of each transcription factor in a given cell [5, 17]. Thus, coregulatory proteins may influence or modulate the activity of a transcription factor through multiple mechanisms [5, 74–76]. In order to fully understand the mechanisms of gene regulation by transcription, we must acquire the knowledge that governs this complex yet extremely important phenomenon. As we learn more and more about the role of binding/folding events in gene regulation by transcription factors, it becomes clearer that highly flexible and dynamic nature of ID regions/domains is an inherent advantage in these molecules that exploits its protein surfaces for critical interactions with various coregulatory proteins in order to achieve desired targets in an efficient and highly specific manner [5]. Under physiological conditions, the ultimate composition of the assembly and kind of induced folding in the ID regions/domain may dictate the final outcome of the signals to be passed by specific transcription factor to the target gene [17]. As we start to understand more about folded functional conformations of these ID stretches and the sequence of events that lead to such folding, we should have answers to many questions that regulate the expression of gene. In addition to protein:protein interactions, for some transcription factors, RNAs are also known to function as cofactors [77], therefore ID-rich transcription factors may offer a platform of RNA binding. It is also important to note that structural flexibility is a common phenomenon in several protein:RNA recognition processes, and these interactions often involve conformational changes in the structure of the RNA, protein, or both [78].

## 6. Summary and Perspectives

No doubt the elucidation of the human genome has provided us an incredible opportunity to find out an immense amount of structural information that may be contained within the human genome, yet our efforts must be devoted to understand how the expression of this genetic information is regulated and how the interactions between the vast array of expressed proteins are controlled. With large data generated from various research groups on protein:protein interactions involving transcription factor and its relationship with target gene regulations, it has become possible to visualize a global view of biological networks. Dynamic macromolecular interactions are key elements in the regulation of many biological systems, particularly in gene regulations by transcription factors [76]. In addition to other factors, the dynamic nature of such protein:protein interactions may be the result of internal conformational dynamics in the constituent molecules [76]. In the recent years, many observations led us to believe that direct protein:protein interaction may be an essential step in realizing properly folded and functionally active structure in their ID region [5]. Though limited, data indicate that the ID sequences may be adopting functionally folded conformation(s) under physiological conditions through these interactions [17]. However, it remains to be determined what kind of functional ordered conformation(s) these ID domains adopt, and whether there are multiple folded conformations generated depending upon the nature of binding partner(s) involved. The resulting structurally modified conformation(s) of these ID regions may further be providing protein surfaces to attract other target molecules, essential for functions [5]. Available knowledge so far on how these ID domains of transcription factors adopt ordered conformation(s) under physiological conditions supports this notion. However, more studies are needed to determine precise mechanisms through which these ID regions/domains acquire a well defined structure. It is now recognized that the conformational flexibility of ID domains/regions is a general feature of most transcription factor proteins, we must take into account their structural features in a network sense as recently described [79]. Since several small molecules as potential drug targets have been found to act by blocking specific protein:protein interactions [18], and that ID regions of transcription factors are known to form the platform for many such protein:protein interactions, a better structural and functional understanding of ID proteins will be a potent tool for drug designing [55]. The utilization of various drug delivery systems such as nanotechnology-based products is anticipated to revolutionize treatments for diseases in future. Thus, rapidly growing field of ID proteins' structural analyses combined with their functional behavior will allow cross-disciplinary researchers the opportunity to design and develop multifunctional approaches to develop better therapeutic tools that will generate novel ideas and help accelerate critical advances in the field of biomedical research.

## References

[1] Dunker AK, Lawson JD, Brown CJ (2001). Intrinsically disordered protein. *Journal of Molecular Graphics and Modelling*.

[2] Dunker AK, Brown CJ, Lawson J (2002). Intrinsic disorder and protein function. *Biochemistry*.

[3] Uversky VN (2002). Natively unfolded proteins: a point where biology waits for physics. *Protein Science*.

[4] Fink AL (2005). Natively unfolded proteins. *Current Opinion in Structural Biology*.

[5] Dyson HJ, Wright PE (2005). Intrinsically unstructured proteins and their functions. *Nature Reviews Molecular Cell Biology*.

[6] Uversky VN, Oldfield CJ, Dunker AK (2005). Showing your ID: intrinsic disorder as an ID for recognition, regulation and cell signaling. *Journal of Molecular Recognition*.

[7] Wright PE, Dyson HJ (1999). Intrinsically unstructured proteins: re-assessing the protein structure-function paradigm. *The Journal of Molecular Biology*.

[8] Eliezer D (2007). Characterizing residual structure in disordered protein states using nuclear magnetic resonance. *Methods in Molecular Biology*.

[9] Dyson HJ, Wright PE (2002). Coupling of folding and binding for unstructured proteins. *Current Opinion in Structural Biology*.

[10] Namba K (2001). Roles of partly unfolded conformations in macromolecular self-assembly. *Genes to Cells*.

[11] Crivici A, Ikura M (1995). Molecular and structural basis of target recognition by calmodulin. *Annual Review of Biophysics and Biomolecular Structure*.

[12] Liu J, Perumal NB, Oldfield CJ, Su EW, Uversky VN, Dunker AK (2006). Intrinsic disorder in transcription factors. *Biochemistry*.

[13] Iakoucheva LM, Brown CJ, Lawson JD, Obradović Z, Dunker AK (2002). Intrinsic disorder in cell-signaling and cancer-associated proteins. *The Journal of Molecular Biology*.

[14] Ward JJ, Sodhi JS, McGuffin LJ, Buxton BF, Jones DT (2004). Prediction and functional analysis of native disorder in proteins from the three kingdoms of life. *The Journal of Molecular Biology*.

[15] Tompa P (2002). Intrinsically unstructured proteins. *Trends in Biochemical Sciences*.

[16] Romero P, Obradovic Z, Dunker AK (2004). Natively disordered proteins: functions and predictions. *Applied Bioinformatics*.

[17] Kumar R, Thompson EB (2003). Transactivation functions of the N-terminal domains of nuclear hormone receptors: protein folding and coactivator interactions. *Molecular Endocrinology*.

[18] Blancafort P, Segal DJ, Barbas CF (2004). Designing transcription factor architectures for drug discovery. *Molecular Pharmacology*.

[19] Durai S, Mani M, Kandavelou K, Wu J, Porteus MH, Chandrasegaran S (2005). Zinc finger nucleases: custom-designed molecular scissors for genome engineering of plant and mammalian cells. *Nucleic Acids Research*.

[20] Beerli RR, Barbas CF (2002). Engineering polydactyl zinc-finger transcription factors. *Nature Biotechnology*.

[21] Jonker HRA, Wechselberger RW, Boelens R, Folkers GE, Kaptein R (2005). Structural properties of the promiscuous VP16 activation domain. *Biochemistry*.

[22] De Guzman RN, Martinez-Yamout MA, Dyson HJ, Wright PE (2004). Interaction of the TAZ1 domain of the CREB-binding protein with the activation domain of CITED2: regulation by competition between intrinsically unstructured ligands for non-identical binding sites. *The Journal of Biological Chemistry*.

[23] Kumar R, Johnson BH, Thompson EB (2004). Overview of the structural basis for transcription regulation by nuclear hormone receptors. *Essays in Biochemistry*.

[24] Ng KP, Potikyan G, Savene ROV, Denny CT, Uversky VN, Lee KAW (2007). Multiple aromatic side chains within a disordered structure are critical for transcription and transforming activity of EWS family oncoproteins. *Proceedings of the National Academy of Sciences of the United States of America*.

[25] Flaugh SL, Lumb KJ (2001). Effects of macromolecular crowding on the intrinsically disordered proteins c-Fos and p27^KIP1^. *Biomacromolecules*.

[26] Campbell KM, Terrell AR, Laybourn PJ, Lumb KJ (2000). Intrinsic structural disorder of the C-terminal activation domain from the bZIP transcription factor Fos. *Biochemistry*.

[27] Kumar R, Thompson EB (2005). Gene regulation by the glucocorticoid receptor: structure:function relationship. *The Journal of Steroid Biochemistry and Molecular Biology*.

[28] Warnmark A, Wikstrom A, Wright APH, Gustafsson J-Å, Härd T (2001). The N-terminal regions of estrogen receptor *α* and *β* are unstructured in vitro and show different TBP binding properties. *The Journal of Biological Chemistry*.

[29] McEwan IJ, Dahlman-Wright K, Ford J, Wright APH (1996). Functional interaction of the c-Myc transactivation domain with the TATA binding protein: evidence for an induced fit model of transactivation domain folding. *Biochemistry*.

[30] Shen F, Triezenberg SJ, Hensley P, Porter D, Knutson JR (1996). Transcriptional activation domain of the herpesvirus protein VP16 becomes conformationally constrained upon interaction with basal transcription factors. *The Journal of Biological Chemistry*.

[31] Uversky VN, Gillespie JR, Fink AL (2000). Why are “natively unfolded” proteins unstructured under physiologic conditions?. *Proteins*.

[32] Kriwacki RW, Hengst L, Tennant L, Reed SI, Wright PE (1996). Structural studies of p21Waf1/Cip1/Sdi1 in the free and Cdk2-bound state: conformational disorder mediates binding diversity. *Proceedings of the National Academy of Sciences of the United States of America*.

[33] Ebert M-O, Bae S-H, Dyson HJ, Wright PE (2008). NMR relaxation study of the complex formed between CBP and the activation domain of the nuclear hormone receptor coactivator ACTR. *Biochemistry*.

[34] Sugase K, Dyson HJ, Wright PE (2007). Mechanism of coupled folding and binding of an intrinsically disordered protein. *Nature*.

[35] De Guzman RN, Goto NK, Dyson HJ, Wright PE (2006). Structural basis for cooperative transcription factor binding to the CBP coactivator. *The Journal of Molecular Biology*.

[36] Zor T, De Guzman RN, Dyson HJ, Wright PE (2004). Solution structure of the KIX domain of CBP bound to the transactivation domain of c-Myb. *The Journal of Molecular Biology*.

[37] Klinkenberg LG, Mennella TA, Luetkenhaus K, Zitomer RS (2005). Combinatorial repression of the hypoxic genes of *Saccharomyces cerevisiae* by DNA binding proteins Rox1 and Mot3. *Eukaryotic Cell*.

[38] Pilpel Y, Sudarsanam P, Church GM (2001). Identifying regulatory networks by combinatorial analysis of promoter elements. *Nature Genetics*.

[39] Holstege FC, Young RA (1999). Transcriptional regulation: contending with complexity. *Proceedings of the National Academy of Sciences of the United States of America*.

[40] Kato M, Hata N, Banerjee N, Futcher B, Zhang MQ (2004). Identifying combinatorial regulation of transcription factors and binding motifs. *Genome Biology*.

[41] Hannenhalli S, Levy S (2002). Predicting transcription factor synergism. *Nucleic Acids Research*.

[42] Guha TD, Stormo GD (2001). Identifying target sites for cooperatively binding factors. *Bioinformatics*.

[43] Wang W, Cherry JM, Nochomovitz Y, Jolly E, Botstein D, Li H (2005). Inference of combinatorial regulation in yeast transcriptional networks: a case study of sporulation. *Proceedings of the National Academy of Sciences of the United States of America*.

[44] Zhong H, Vershon AK (1997). The yeast homeodomain protein MAT*α*2 shows extended DNA binding specificity in complex with Mcm1. *The Journal of Biological Chemistry*.

[45] Halfon MS, Carmena A, Gisselbrecht S (2000). Ras pathway specificity is determined by the integration of multiple signal-activated and tissue-restricted transcription factors. *Cell*.

[46] Anfinsen CB, Scheraga HA (1975). Experimental and theoretical aspects of protein folding. *Advances in Protein Chemistry*.

[47] Baskakov I, Bolen DW (1998). Forcing thermodynamically unfolded proteins to fold. *The Journal of Biological Chemistry*.

[48] Lumry R, Biltonen R, Brandts JF (1966). Validity of the “two-state” hypothesis for conformational transitions of proteins. *Biopolymers*.

[49] Kumar R, Baskakov IV, Srinivasan G, Bolen DW, Lee JC, Thompson EB (1999). Interdomain signaling in a two-domain fragment of the human glucocorticoid receptor. *The Journal of Biological Chemistry*.

[50] Baskakov IV, Kumar R, Srinivasan G, Ji Y-S, Bolen WD, Thompson EB (1999). Trimethylamine *N*-oxide-induced cooperative folding of an intrinsically unfolded transcription-activating fragment of human glucocorticoid receptor. *The Journal of Biological Chemistry*.

[51] Kumar R, Lee JC, Bolen DW (2001). The conformation of the glucocorticoid receptor AF1/tau1 domain induced by osmolyte binds co-regulatory proteins. *The Journal of Biological Chemistry*.

[52] Bain DL, Franden MA, McManaman JL, Takimoto GS, Horwitz KB (2000). The *N*-terminal region of the human progesterone A-receptor. Structural analysis and the influence of the DNA binding domain. *The Journal of Biological Chemistry*.

[53] Warnmark A, Wikstrom A, Wright APH, Gustafsson J-Å, Härd T (2001). The *N*-terminal regions of estrogen receptor *α* and *β* are unstructured in vitro and show different TBP binding properties. *The Journal of Biological Chemistry*.

[54] Iakoucheva LM, Radivojac P, Brown CJ (2004). The importance of intrinsic disorder for protein phosphorylation. *Nucleic Acids Research*.

[55] Romero PR, Zaidi S, Fang YY (2006). Alternative splicing in concert with protein intrinsic disorder enables increased functional diversity in multicellular organisms. *Proceedings of the National Academy of Sciences of the United States of America*.

[56] Baskakov I, Wang A, Bolen DW (1998). Trimethylamine-N-oxide counteracts urea effects on rabbit muscle lactate dehydrogenase function: a test of the counteraction hypothesis. *Biophysical Journal*.

[57] Liu Y, Bolen DW (1995). The peptide backbone plays a dominant role in protein stabilization by naturally occurring osmolytes. *Biochemistry*.

[58] Baldwin RL, Rose GD (1999). Is protein folding hierarchic? I. Local structure and peptide folding. *Trends in Biochemical Sciences*.

[59] Yancey PH, Clark ME, Hand SC, Bowlus RD, Somero GN (1982). Living with water stress: evolution of osmolyte systems. *Science*.

[60] Burg MB (1995). Molecular basis of osmotic regulation. *The American Journal of Physiology*.

[61] Bolen DW (2001). Protein stabilization by naturally occurring osmolytes. *Methods in Molecular Biology*.

[62] Bolen DW (2004). Effects of naturally occurring osmolytes on protein stability and solubility: issues important in protein crystallization. *Methods*.

[63] Bolen DW, Baskakov IV (2001). The osmophobic effect: natural selection of a thermodynamic force in protein folding. *The Journal of Molecular Biology*.

[64] Auton M, Bolen DW (2005). Prediction the energetics of osmolyte-induced protein folding/unfolding. *Proceedings of the National Academy of Sciences of the United States of America*.

[65] Holthauzen LMF, Bolen DW (2007). Mixed osmolytes: the degree to which one osmolyte affects the protein stabilizing ability of another. *Protein Science*.

[66] Rosgen J, Pettitt BM, Bolen DW (2005). Protein folding, stability, and solvation structure in osmolyte solutions. *Biophysical Journal*.

[67] Street TO, Bolen DW, Rose GD (2006). A molecular mechanism for osmolyte-induced protein stability. *Proceedings of the National Academy of Sciences of the United States of America*.

[68] Hochachka PW, Somero GN (2002). *Biochemical Adaptation: Mechanism and Process in Physiological Evolution*.

[69] Yancey PH, Somero GN (1980). Methylamine osmoregulatory solutes of elasmobranch fishes counteract urea inhibition of enzymes. *Journal of Experimental Zoology*.

[70] Mashino T, Fridovich I (1987). Effects of urea and trimethylamine-N-oxide on enzyme activity and stability. *Archives of Biochemistry and Biophysics*.

[71] Kumar R, Serrette JM, Khan SH, Miller AL, Thompson EB (2007). Effects of different osmolytes on the induced folding of the N-terminal activation domain (AF1) of the glucocorticoid receptor. *Archives of Biochemistry and Biophysics*.

[72] Tanaka M, Machida Y, Niu S (2004). Trehalose alleviates polyglutamine-mediated pathology in a mouse model of Huntington disease. *Nature Medicine*.

[73] Minezaki Y, Homma K, Kinjo AR, Nishikawa K (2006). Human transcription factors contain a high fraction of intrinsically disordered regions essential for transcriptional regulation. *The Journal of Molecular Biology*.

[74] Mark W-Y, Liao JC, Lu Y (2005). Characterization of segments from the central region of BRCA1: an intrinsically disordered scaffold for multiple protein-protein and protein-DNA interactions?. *The Journal of Molecular Biology*.

[75] Levy Y, Onuchic JN, Wolynes PG (2007). Fly-casting in protein-DNA binding: frustration between protein folding and electrostatics facilitates target recognition. *Journal of the American Chemical Society*.

[76] Bernadó P, Blackledge M, Sancho J (2006). Sequence-specific solvent accessibilities of protein residues in unfolded protein ensembles. *Biophysical Journal*.

[77] Lanz RB, McKenna NJ, Onate SA (1999). A steroid receptor coactivator, SRA, functions as an RNA and is present in an SRC-1 complex. *Cell*.

[78] Tompa P, Csermely P (2004). The role of structural disorder in the function of RNA and protein chaperones. *FASEB Journal*.

[79] Csermely P (2008). Creative elements: network-based predictions of active centres in proteins and cellular and social networks. *Trends in Biochemical Sciences*.

